# Preparation and Antibacterial Activity of Nano Copper Oxide- Loaded Zeolite 10X

**DOI:** 10.3390/ijms23158421

**Published:** 2022-07-29

**Authors:** Yang Ma, Jin Hou

**Affiliations:** 1 Key Laboratory of Marine Chemistry Theory and Technology, Ministry of Education, College of Chemistry and Chemical Engineering, Ocean University of China, Qingdao 266100, China; jhhxhg@163.com; 2 Shandong Key Laboratory of Corrosion Science, Institute of Oceanology, Chinese Academy of Sciences, Qingdao 266100, China

**Keywords:** antibacterial activity, copper oxide nanosheet, zeolite, concentration, particle size

## Abstract

Copper oxide nanosheet-loaded zeolite 10X nanocomposites (CuO-zeolite NCs) were successfully prepared by modifying zeolite 10X with CuSO_4_ aqueous solution. The formation of copper oxide nanosheets on the surface of zeolite 10X was observed by SEM. The thickness of CuO nanosheets was about 30–40 nm, and the width ranged from 200 nm to 300 nm. The XRD patterns showed that the new diffraction peaks of copper oxide appeared at 35.6° and 38.8°. According to the XPS results, the Cu 2p_3/2_ and Cu 2p_1/2_ peaks in CuO-zeolite NC were centered at 934.1 eV and 953.8 eV, which could be attributed to Cu(II). The EDS analysis revealed that the energy spectra of calcium gradually decreased as the copper ion concentration increased during the preparation of CuO-zeolite NCs. Meanwhile, the energy spectra of copper increased gradually, and the highest content of copper in CuO-zeolite NCs reached 22.35 wt.%. The BET surface areas of zeolite 10X and CuO-zeolite NCs were 587 and 363 m^2^/g, respectively, based on the N_2_ adsorption–desorption experiment. The antibacterial activities of CuO-zeolite NC were evaluated using *Escherichia coli* (*E. coli*) and *Staphylococcus aureus* (*S. aureus*). The antibacterial activities were related to both copper ion content in CuO-zeolite NCs and the particle size of copper oxide. The results showed that nano CuO-loaded zeolite 10X inhibited the activity of *E. coli* and *S. aureus*. CuO-zeolite NCs are expected to be further used in antifouling coating.

## 1. Introduction

Nanocomposite materials have attracted significant interest due to their unique properties, which are absent in conventional composites [1]. The specific properties of nanocomposite materials differ from those of the bulk compounds, which have led to further studies of nanocomposites in many different fields [2]. Metal oxide nanoparticles have attracted great interest, due to their unique chemical and physical properties.

Copper oxide nanoparticles (CuO NPs) have been extensively studied due to their superior physical and chemical performance in applications such as catalysis [3], sensors [4,5], thermosensing and conducting materials [6], solar cells [7], environmental remediation [8], and textile product manufacturing [9]. The United States Environmental Protection Agency (US EPA) have approved copper and its alloys as solid surface materials to kill 99.9% of all bacteria on surfaces within 2 h of contact [10]. CuO NPs are cost-effective, non-toxic, and abundant. They exhibit unique physicochemical properties due to their quantum size effect and high specific surface area, which increase the biological and chemical activity of the material. Another important feature is the ability to target various types of bacterial structures. In addition, they can be easily blended with many polymers and other substrates, prompting several studies in this area [11,12,13,14].

Among different kinds of substrates, zeolites are regarded as promising materials due to their high ion-exchange capacity, thermal stability, high surface area, and eco-friendly features [15,16,17]. A CuO NP-loaded zeolite has been fabricated for different applications [18,19]. To the best of our knowledge, different methods for the preparation of CuO NP-loaded zeolites have been reported. The role of CuO NPs as antibacterial agents to inhibit bacterial growth was studied. Ultra-small CuO NPs were synthesized using a mechanochemical method with two different Cu-containing precursors (i.e., CuSO_4_·5H_2_O and CuCl_2_·2H_2_O). Their antibacterial activity was determined by determining the minimum inhibitory concentrations and using disc diffusion and colony-counting methods. The CuCl_2_·2H_2_O-derived nanoparticles showed higher antibacterial activity than CuSO_4_·5H_2_O-derived nanoparticles [20]. The actinomycete-mediated biosynthesis of CuO NPs and their antibacterial activity against selected human and fish pathogens were reported [21]. Alswat studied the preparation of CuO NPs-loaded zeolite using a simple and green precipitation method, and the antibacterial activities against *Bacillus subtilis B29* and *Salmonella choleraesuis* were measured by inhibition zone [22]. Several methods, such as sol-gel, thermal oxidation, and sonochemical tests, have been proposed for the synthesis of CuO NPs. Among all the proposed methods for the synthesis of CuO NPs, the precipitation method is simple and easy to operate.

Zeolite 10X is one of the most widely used due to its highest cation exchange capacity. The cations in the pores of zeolite 10X can be exchanged by different cations, such as copper ions. Therefore, zeolite exhibits antibacterial activity mediated via cation exchange with copper ions. Antibacterial zeolites have achieved great significance compared with conventional antibacterial agents. The incorporation of antimicrobial metallic ions within the zeolite framework facilitates their controlled release and prevents concentration-dependent toxicity [23].

In this work, a simple method was used to synthesize CuO nanosheets on the surface of zeolite 10X to obtain CuO-zeolite NCs. The samples were characterized by X-ray diffraction (XRD), scanning electron microscopy (SEM), X-ray photoelectron spectroscopy (XPS), energy dispersion spectroscopy (EDS), and N_2_ adsorption–desorption experiments. In the absence of studies describing the antibacterial activities related to copper ion content and particle size of CuO, the antibacterial activities against *E. coli* and *S. aureus* were evaluated via the spread-plate method.

## 2. Results and Discussion

### 2.1. Characterization of Zeolite

**Figure 1:**
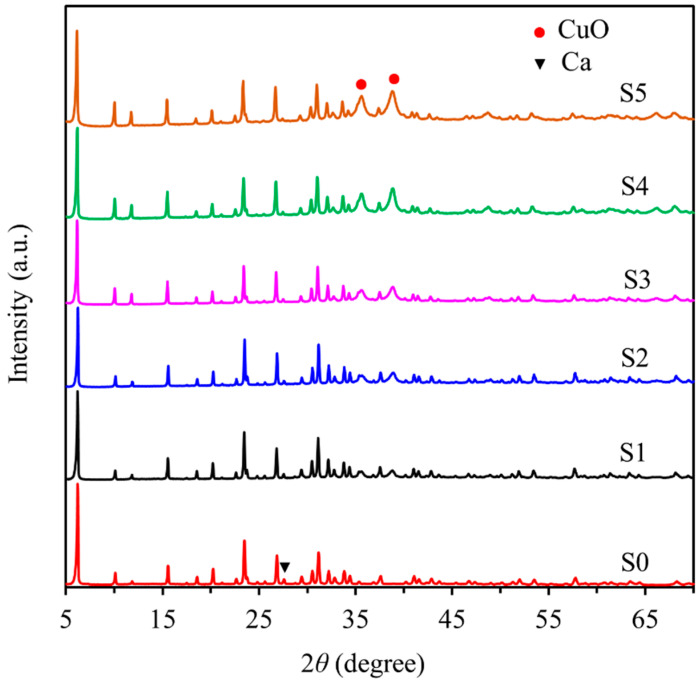
XRD patterns of samples: (S0) zeolite 10X; (S1–S5) CuO-zeolite NCs.

The effects of copper ion concentration (0.010 M, 0.020 M, 0.040 M, 0.060 M and 0.090 M) on CuO-zeolite NC were studied. The XRD patterns of zeolite 10X and CuO-zeolite NCs are shown in Figure 1 (S0–S5). The diffraction peaks shown in Figure 1 (S0) correspond to the characteristic peaks of zeolite 10X. When the concentration of copper ion was 0.010 M, the new and weak diffraction peaks began to appear at 35.6° and 38.8°, respectively, as shown in Figure 1 (S1), which correspond to the characteristic peaks of CuO [22]. The diffraction peaks of CuO gradually increased and became obvious with the increase in copper ion concentrations from 0.020 to 0.060 M, as shown in Figure 1 (S2–S4). When the concentration of copper ions was 0.090 M, the diffraction peaks of CuO showed no clear improvement, as shown in Figure 1 (S5). Meanwhile, the diffraction peak of Ca at 27.6° gradually decreased with the increase in copper ion concentrations [24]. The diffraction peaks between 35° and 39° indicated the formation of oxides and belonged to CuO [25].

**Figure 2:**
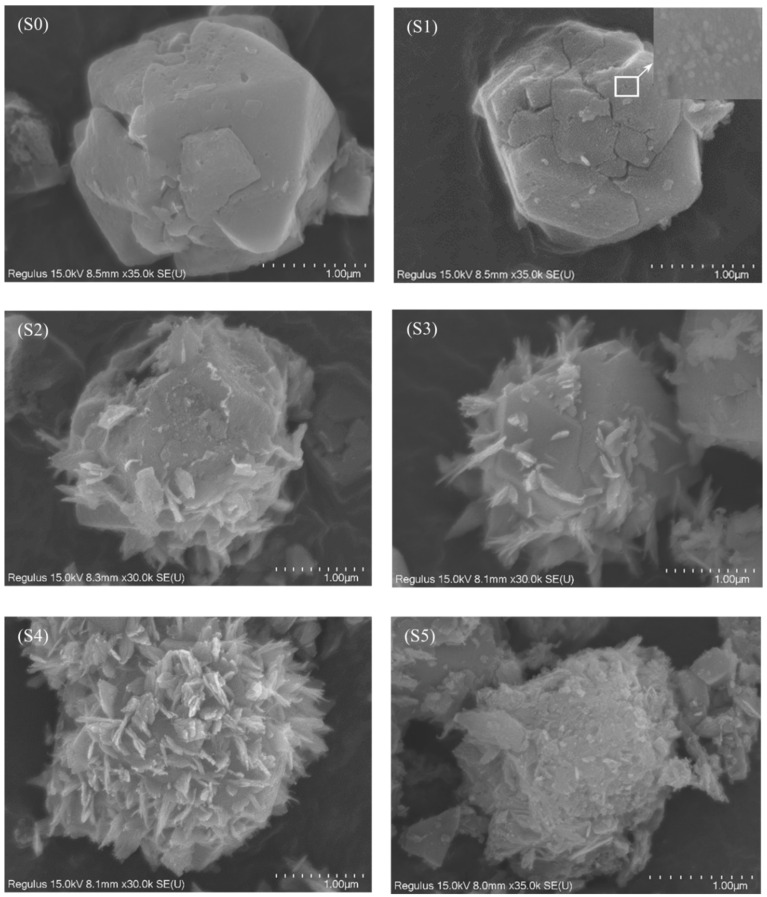
SEM images of samples: (S0) zeolite 10X; (S1–S5) CuO-zeolite NCs.

Figure 2 shows SEM micrographs of zeolite 10X and CuO-zeolite NCs. The image in Figure 2 (S0) shows irregular and grainy zeolite 10X. The surfaces of CuO-zeolite NCs illustrated in Figure 2 (S1–S5) differed from those of zeolite 10X. A small amount of CuO nanosheets were formed on the surface of sample S1 when the concentration of Cu ions was 0.010 M, and the particle size was in the range of 10–20 nm, suggesting tiny CuO nanosheets. As shown in Figure 2 (S2), a small amount of CuO nanosheets formed when the concentration of Cu ions reached 0.020 M, which suggested formation of CuO nanosheets. As shown in Figure 2 (S3, S4), additional CuO nanosheets were detected clearly when the concentrations were 0.040 and 0.060 M. The thickness of CuO nanosheets was about 30–40 nm, and the width ranged from 200 nm to 300 nm, indicating the formation of CuO nanosheets. CuO nanosheets agglomerated when the concentration of he Cu ion was 0.090 M, which greatly reduced the properties of CuO-zeolite NCs.

**Figure 3:**
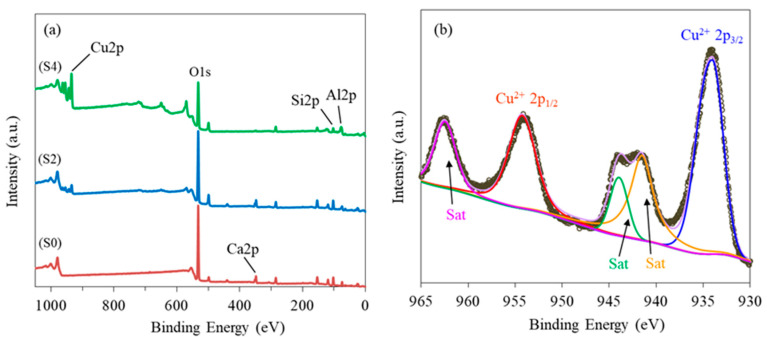
XPS spectra of S0, S2 and S4 samples: (**a**) survey; (**b**) Cu 2p.

The XPS analysis of zeolite 10X and CuO-zeolite NCs are shown in Figure 3a,b. The survey spectrum in Figure 3a indicates that zeolite 10X (S0) was mainly composed of Si, Al, Ca, and O, and CuO-zeolite NCs (S2, S4) contained mostly Cu, Si, Al, and O. The increase in Cu ion concentration resulted in the emergence of Cu peaks, while the intensity of the Ca peak declined, indicating the replacement of Ca ions by Cu ions. The high-resolution XPS spectrum of Cu 2p is shown in Figure 3b. The Cu 2p_3/2_ and Cu 2p_1/2_ peaks centered at 934.1 eV and 953.8 eV, respectively, and were attributed to Cu(II) [22,26]. The presence of only Cu(II) was verified by XPS.

**Figure 4:**
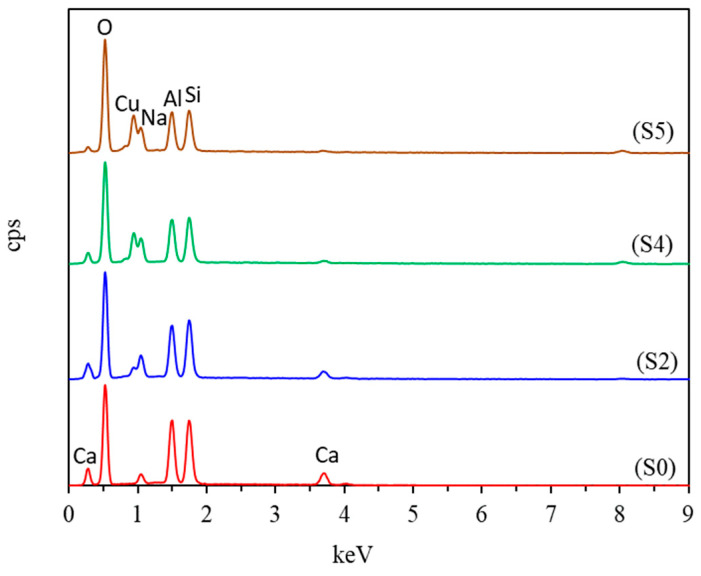
EDS of samples: (S0) zeolite 10X; (S2, S4 and S5) CuO-zeolite NCs.

The elemental analysis of the prepared zeolite 10X and CuO-zeolite NCs was performed via EDS (Figure 4). Figure 4 (S0) shows that zeolite 10X is mainly composed of Si, Al, Ca, and O elements. Figure 4 (S2, S4 and S5) shows that the energy spectrum of Ca declined with the increasing Cu ion concentration. The Ca content in S0, S2, S4, and S5 was 8.40 wt.%, 5.00 wt.%, 1.57 wt.%, and 0.94 wt.%, respectively. Meanwhile, the Cu energy spectrum appeared and increased gradually. The Cu content in S2, S4, and S5 was 6.61 wt.%, 19.18 wt.%, and 22.35 wt.%, respectively, indicating he replacement of Ca ions by Cu ions.

**Figure 5:**
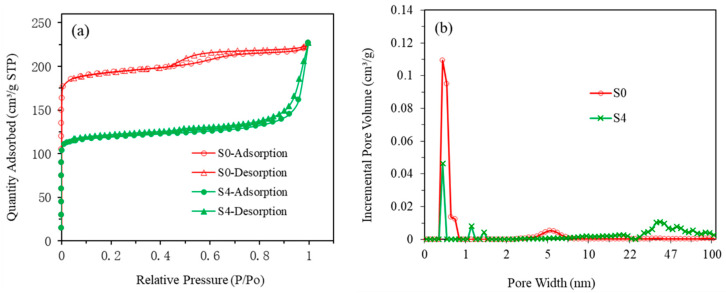
The adsorption of N_2_ (**a**) and the pore size distribution curve (**b**) of samples (S0 and S4).

The adsorption of N_2_ and the pore size distributions of zeolite 10X and CuO-zeolite NC (S4) were measured using N_2_ adsorption–desorption isotherms, and the results are shown in Figure 5. Before gas adsorption measurements, zeolite 10X and sample S4 were degassed at 200 °C for 500 min under a vacuum. As shown in Figure 5a, the adsorption of samples S0 and S4 reached 230 cm^3^/g at a relative pressure of 1.0. The N_2_ adsorption–desorption isotherm of sample S0 generated desorption hysteresis and presented a hysteresis loop, which indicates the presence of mesopores in the material. Sample S0 revealed a Langmuir isotherm (type-I) characteristic. The N_2_ adsorption of sample S4 exhibited the features of a type-II isotherm. The incremental pore volume of sample S4 was smaller than that of sample S0 in the main pore size range, as shown in Figure 5b. The single point adsorption total pore volumes of samples S0 and S4 were 0.34 and 0.23 cm^3^/g, respectively. The BET surface areas of sample S0 and S4 were 587 and 363 m^2^/g, respectively. From sample S0 to S4, the type of isotherms changed. As the relative pressure continued to increase, multilayer adsorption gradually formed in S4, and when the saturated vapor pressure was reached, there were infinite adsorption layers, resulting in a sharp rise of quantity adsorbed at the end of the curve. The reason can be attributed to the growth of copper oxide on the surface and in the pores of zeolite, which led to the filling or blocking of some pores. This could be verified from the reduction of BET surface area and pore volume of sample S4. Sample S0 produced desorption hysteresis and presented a hysteresis loop that belonged to H4, which was due to the existence of both micropores and mesopores, as shown in Figure 5b. Sample S4 presented hysteresis loop that belonged to H3, which was related to the accumulation of sheet particles. The reason for hysteresis loop of type H3 may be that macropores were formed during the formation of copper oxide nanosheets, as shown in Figure 5b. The adsorption saturation was not reached in the region of high relative pressure.

### 2.2. Antibacterial Activity of CuO-Zeolite NCs

**Figure 6:**
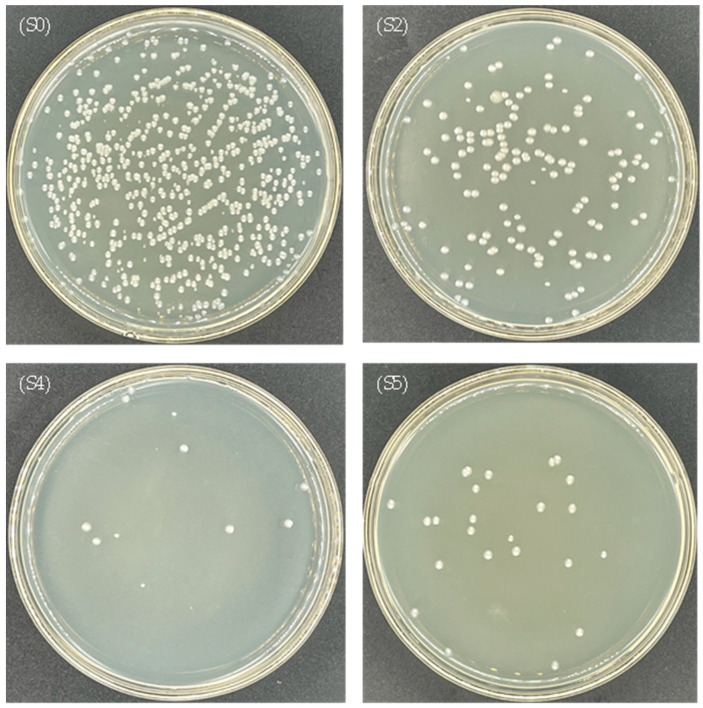
Photographs of the growth of *E. coli* on a petri-dish: (S0) control sample, (S2, S4, S5) experimental samples.

**Figure 7:**
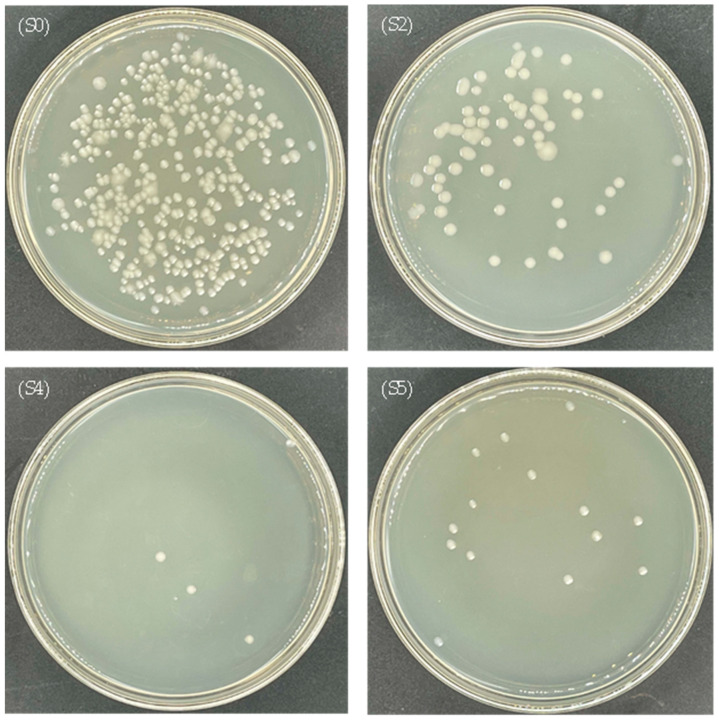
Photographs of the growth of *S. aureus* on a petri-dish: (S0) control sample, (S2, S4, S5) experimental samples.

The antibacterial efficiencies of CuO-zeolite NCs (S2, S4 and S5) were evaluated using *E. coli* and *S. aureus*. After incubation for 20 h at 37 °C, the numbers of bacteria in the experimental samples were significantly more reduced than in the control samples, as shown in Table 1. The growth of *E. coli* and *S. aureus* on the petri-dish is illustrated in Figure 6 and Figure 7. As shown in Figure 6, the number of live bacteria on the petri-dish was 580 in the control sample (S0). The numbers of live bacteria on the petri-dish were 143, 11 and 23 in S2, S4, and S5 experimental samples, respectively. The antibacterial efficiencies of samples S2, S4, and S5 were 75.3%, 98.1%, and 96.0% against *E. coli*, respectively. As shown in Figure 7, the number of live bacteria was 380 in the control sample (S0). The numbers of live bacteria were 66, 4, and 14 in the experimental samples (S2, S4, and S5, respectively). The antibacterial efficiencies of sample S2, S4, and S5 against *S. aureus* reached 82.6%, 98.9%, and 96.3%, respectively. The antibacterial efficiencies of samples S2 and S4 improved with the increase in the Cu ion levels under the same dosage of the samples. The antibacterial efficiencies of sample S2 against *E. coli* and *S. aureus* were 75.3% and 82.6%, respectively. In the case of sample S4, the antibacterial efficiencies against *E. coli* and *S. aureus* exceeded 98%. However, the antibacterial activity of sample S5 was lower than that of sample S4, although the Cu content in sample S5 was higher than that of sample S4, possibly due to the high surface-to-volume ratio of nanosheets in sample S4, which increased the release of ions with antibacterial activity. The agglomeration of nanosheets in sample S5 reduced the release of Cu ions. The SEM images show that the morphological features of sample S5 differed from those of other samples (S2–S4), since individual nanosheets were absent.

Under the same conditions, by comparing the antibacterial effect on *E. coli* and *S. aureus*, the antibacterial ability of CuO-zeolite NCs against the two strains was basically the same, but the antibacterial efficiency against *S. aureus* was slightly stronger. When the Cu content increased from 6.61 wt.% to 19.18 wt.%, the antibacterial efficiency against *E. coli* and *S. aureus* increased by 30% and 20%, respectively. When the Cu content increased from 19.18 wt.% to 22.35 wt.%, the antibacterial efficiency against the two strains decreased by 2–3%. In the case of low sample concentration (1.3 mg/mL), the antibacterial effects were still better than those of reported CuO NPs [20]. The results prove that the CuO NP-loaded zeolite 10X exhibits a strong bactericidal effect.

Cu^2+^ ions released from nanosheets may adhere to the negatively-charged bacterial cell wall, and its disruption leads to protein denaturation and cell death [27]. Another proposed mechanism involves destruction of the bacterial cell membrane and enzyme activity by Cu^2+^ ions. The indirect effect caused by the changes in the surrounding ionic environment also determines the antibacterial effectiveness of NP metals [27,28].

## 3. Materials and Methods

### 3.1. Materials

Zeolite 10X (2–3 μm) was obtained from Macklin (Shanghai, China). CuSO_4_·5H_2_O, NaOH, and NaCl (AR) were purchased from Shanghai Sinopharm Chemical Reagent Co., Ltd. (Shanghai, China). A nutrient broth and nutrient agar (BR) were supplied by Beijing Land Bridge Technology Co., Ltd. (Beijing, China). A petri-dish was ordered from Qingdao Hope Bio-Technology Co., Ltd. (Qingdao, China). *Escherichia coli* (*E. coli*) and *Staphylococcus aureus* (*S. aureus*) were kindly provided by the microbiology laboratory at the College of Marine Life Sciences, Ocean University of China (Qingdao, China).

### 3.2. Preparation of CuO-Zeolite NCs

First, 1.0 g of zeolite 10X (signed S0) was added to different concentrations of copper ion solution (0.010 M, 0.020 M, 0.040 M, 0.060 Mm and 0.090 M). The ion-exchange reaction occurred between Cu^2+^ ions and Ca^2+^ ions in zeolite 10X after the five solutions were stirred evenly for 2 h at 80 °C to obtain the Cu^2+^ ion-loaded zeolite 10X. For complete oxidation of the bound Cu^2+^, 2 M of sodium hydroxide solution was added to the above suspension until the pH was about 12. The suspension was stirred at 80 °C for 2 h. The color of the mixture changed from blue to brown and black, suggesting the formation of copper oxide. CuO-zeolite NCs were obtained via filtration, thoroughly washed with deionized water, and dried at 80 °C overnight. The resulting CuO-zeolite NCs were designated as S1, S2, S3, S4 and S5 according to the concentration of the copper ion solution from low to high levels. The formation of CuO-zeolite NCs is represented by the following reactions [22]:

Ca-Zeolite + Cu^2+^ → Cu^2+^-Zeolite

Cu^2+^-Zeolite + NaOH → Na-Zeolite + Cu(OH)_2_

Na-Zeolite + Cu(OH)_2_ → CuO-zeolite NCs

### 3.3. Characterization

Structural features of zeolite 10X and CuO-zeolite NCs were evaluated by XRD (Bruker D8-Advance, Karlsruhe, Germany) using λ = 1.5418 Å Cu K_α_ radiation in the wide-angle range of 2*θ* (5–70°) at a scan speed of 6°/min. The surface morphology of zeolite 10X and CuO-zeolite NCs was identified using scanning electron microscopy (SEM, S-4800, Tokyo, Japan). Elemental analyses were performed by XPS (ESCALAB 250XI, Waltham, MA, USA) and EDS (E-max, Tokyo, Japan). The pore structures of zeolite 10X and CuO-zeolite NCs were characterized by N_2_ adsorption–desorption isotherms using a static volumetric sorption analyzer (ASAP2020, Norcross, GE, USA) at 77 K. An MLS-3750 autoclave sterilizer (Sanyo, Osaka, Japan) was used for sterilization of utensils. An HZQF160 shaking incubator (Beijing, China) was used for bacterial culturing.

### 3.4. Antibacterial Activity Assays

The antibacterial activity was tested by the spread-plate method. Nutrient broth (19.0 g) was added to 1000 mL of deionized water. The liquid culture medium was obtained after the solution was sterilized at 121 °C for 15 min. Nutrient agar (33.0 g) was added to 1000 mL of deionized water and sterilized at 121 °C for 15 min. About 30 mL of the nutrient agar solution was added to a sterile Petri dish and cooled until solidification to obtain the solid culture medium.

*E. coli* and *S. aureus* were recovered for 24 h at 37 °C. A small amount of recovered bacterial strain and 15 mL of liquid culture medium were transferred into the same sterile test tube, which was then sealed. The bacterial culture solution was obtained after the mixture was incubated in a shaking incubator for 24 h at 37 °C. The bacterial culture was diluted to a concentration of 10^8^ CFU/mL.

Sterile CuO-zeolite NCs (20 mg), 15 mL of liquid culture medium, and 200 μL of bacteria culture solution were added to the same sterile test tube and sealed to obtain a bacterial suspension of 1.3 mg/mL of the CuO-zeolite NC sample. The bacterial suspension was incubated for 18 h at 37 °C and marked as an experimental sample. According to the same method, the control sample was obtained by replacing CuO-zeolite NCs with sterile zeolite 10X (20 mg). The control and experimental sample solutions were, respectively, diluted 10-fold with sterile normal saline. Finally, 200 µL of the bacterial suspension was lightly coated on a solid culture medium until the bacterial suspension was fully absorbed, and three parallel experiments were performed with each sample. After incubation for 20 h at 37 °C, the growth of bacteria on petri-dishes was photographed with a camera and compared.

The antibacterial efficiency (*A*) was evaluated according to the following equation [29]:

(1) $$A=\frac{N_{0}-N_{i}}{N_{0}}\times 100\%$$

where *N*_0_ and *N_i_* represent the numbers of living bacterial colonies on the petri-dish, corresponding to the control and the experimental samples, respectively.

## 4. Conclusions

CuO-zeolite NCs were successfully prepared by modifying zeolite 10X, and the antibacterial activities against *E. coli* and *S. aureus* were studied. A simple method was used to synthesize CuO nanosheets on the surface of zeolite 10X. The XPS results confirmed the existence of only Cu(II). The EDS analysis indicated that the energy spectra of calcium gradually decreased with increasing Cu ion concentration, which proves that Ca ions were replaced by Cu ions. Zeolite 10X played an important role in hosting CuO and regulating its release, due to its porous structure and large BET surface area.

The content of Cu^2+^ ions in sample S2 were 6.61 wt.%, and the antibacterial efficiencies against *E. coli* and *S. aureus* were 75.3% and 82.6%, respectively. The level of Cu^2+^ ions in sample S4 were 19.18 wt.%, and the antibacterial efficiencies against *E. coli* and *S. aureus* exceeded 98%. The content of Cu^2+^ ions in sample S5 increased to 22.35 wt.%, while the antibacterial efficiencies against the two bacterial strains decreased with respect to sample S4. CuO nanosheets agglomerated and did not appear in nano form on the surface of sample S5, resulting in inhibition of the release of ions with antibacterial activity. Therefore, the antibacterial activity is related to both Cu^2+^ ion levels and CuO particle size. The antibacterial tests indicated that nano CuO-loaded zeolite 10X is effective against *E. coli* and *S. aureus*. CuO-zeolite NCs have potential applications in antifouling coating.

## Figures and Tables

**Table 1:** Antibacterial activities of CuO-zeolite NCs.

Bacterial Strains	*E. coli*				*S. aureus*			
Samples	S0	S2	S4	S5	S0	S2	S4	S5
Numbers of living bacterial colonies	580	143	11	23	380	66	4	14
*A*/%	/	75.3	98.1	96.0	/	82.6	98.9	96.3

## Data Availability

Data are available upon request.
